# Is Ecological Birdwatching Tourism a More Effective Way to Transform the Value of Ecosystem Services?—A Case Study of Birdwatching Destinations in Mingxi County, China

**DOI:** 10.3390/ijerph182312424

**Published:** 2021-11-25

**Authors:** Tianle Liu, Li Ma, Linsong Cheng, Yilei Hou, Yali Wen

**Affiliations:** School of Economics and Management, Beijing Forestry University, Beijing 100083, China; m13051856877@163.com (T.L.); mali1127@bjfu.edu.cn (L.M.); chenglinsong@bjfu.edu.cn (L.C.)

**Keywords:** recreation value, ecotourism, ecological birdwatching, eco-birdwatcher, general ecotourism, general ecotourist, Travel Cost Interval Analysis (TCIA), ecosystem services, China

## Abstract

Ecological birdwatching tourism is an ecological product and an essential part of ecotourism, and the realization of its recreation value is crucial for improving human well-being, and realization of the local benefits of ecosystem services for areas focused on biodiversity conservation, especially in bird species. In this study, we use travel cost interval analysis, one of the travel cost derived models featuring more easily satisfied assumptions and less limited data, to evaluate the recreation value of the ecological bird-watching tourism destination, and compare it with the general ecotourism, of Mingxi County destination in China. The results show that, firstly, the per capita recreation value of eco-birdwatching is 3.9 times that of general eco-tourism, its per capita social benefit is three times that of general eco-tourism, and its per capita economic benefit is 4.5 times that of general eco-tourism. Secondly, compared with general ecotourists, the per capita travel costs of eco-birdwatchers are higher, and there were significant statistical differences in the expenses for catering, tickets, shopping, opportunity cost, and total travel expenses between these two groups. Thirdly, in comparison with general ecotourists, the marginal cost of an individual eco-birdwatcher is higher, and the travel intention of an eco-birdwatcher is more robust at the same cost level. The price of a single eco-birdwatcher is higher under the same travel intention demand level. In short, the ecological bird-watching industry has a higher marginal value than general eco-tourism and has higher social, economic, and ecological benefits, bringing a higher level of development for the local tourism industry.

## 1. Introduction

Ecosystem services effectively strengthen the relationship between ecosystem functions and human well-being [1]. Costanza (1998) proposed that the valuation of recreational destinations is a vital accounting element in ecosystem services [2]. For areas focused on biodiversity conservation, especially in bird species, ecological tourism, including ecological birdwatching, is an essential natural capital and form of ecosystem services. Since the 18th National Congress of the Communist Party of China, ecological conservation has been included in the overall layout of “the five-pronged approach to building socialism with Chinese characteristics”, which shows that it has risen to the height of the national ecological security strategy. Then in April 2021 it was clearly proposed that the ecological product value accounting system be initially established by 2025. The theme of the upcoming 15th Conference of the Parties to the United Nations Convention on Biological Diversity (CBD COP15) is “Ecological Civilization: Building a Community of Life on Earth”. At the same time, China has carried out projects with mountains, rivers, lakes, forests, farmlands, and grasslands to integrate their protection and effective restoration and actively promote the construction of key ecological projects and biodiversity conservation. With this solid ecological protection and restoration work, China’s ecological environment quality continues to improve.

In China, the main measure of protecting endangered bird species is to establish protected areas [3]. Developing ecotourism has proven effective in areas where human-bird interaction is high [4]. One of the countries with the most abundant bird population worldwide, there are 1445 species in China [5], and four of the nine migratory routes in the world run through the whole territory [6]. The vigorous development of bird protection work in the past 40 years has significantly increased the population of some rare bird species. In recent years, with the significant enrichment of people’s material life, the improvement of their comprehensive quality of life, and the strengthening of their awareness of environmental protection, the number and frequency of people’s ecotourism trips has risen [7], and the concept of “eco-product” has emerged as the times require. As a new form of ecotourism with a sharp increase audience size in recent years, birdwatching tourism has attracted much attention. Various bird protection organizations, birdwatching associations, bird photography associations, and other birdwatching-related organizations have been accompanied by the development of birdwatching tourism and the evolution of the industry [8].

Ecotourism involves responsible travel to natural areas that conserve the environment, sustaining the well-being of the local people and wild animals. It can improve the well-being of local community residents and tourists [9], especially in helping tourists to achieve suitable physical, mental and spiritual states, thus promoting the development of public health [10]. Ecological birdwatching tourism, as a cultural service [11], is generally regarded as one of the most critical manifestations of ecotourism, featuring less investment costs, higher dependence, and fewer side effects affecting the destination environment, with ecotourists having a particular attachment to birdwatching activities and the production of natural empathy [12]. In our study, we divided ecotourism into two parts: ecological birdwatching and general ecotourism. Ecological birdwatching is a kind of tourism behavior that takes birdwatching as the main purpose and takes the activities or habitats of wild birds to “watch” or “shoot” or both without affecting their normal life and minimizing the impact on their habitats; while general ecotourism is that larger part of ecotourism lying behind eco-birdwatching tourism. Eco-birdwatchers are those tourists who pursue eco-birdwatching activities defined above; at the same time, general ecotourists are tourists in addition to eco-birdwatchers. The eco-birdwatching industry is an important part of ecotourism. As an ecological product, the realization of birdwatching’s value is of great significance for the achievement of ecological, social, and economic benefits within specific regional units.

## 2. Literature Review

The travel cost method (TCM) has developed into many more derived models since 1959 [13], each of them with its limitations and data type. Zonal TCM (ZTCM) was a term proposed by Clawson and Knetsch in 1966 [14]. Visitors from the same region are required to have similar travel costs supported by a sufficiently large sample size. Individual TCM (ITCM) was proposed by Brown et al. [15] in 1973, and requires that the number of trips made by tourists be discrete. The Advanced Individual Travel Cost Method (AITCM) is a newer model proposed by scholars using advanced econometric models to make up for theoretical defects in the original ITCM model, but its limitations have not yet been fully resolved [16]. Following the hypothesis of “rational economic man”, Chinese scholars have put forward the improved Travel Cost Interval Analysis (TCIA), which has easy-to-meet data requirements and is based on the assumption that every tourist will pursue the maximization of income or the minimization of cost. Every tourist is willing to travel at a price lower than the current cost, so the travel cost of the sample is divided into several reasonable intervals to fit the travel demand curve of a single tourist, in which the horizontal axis represents the individual travel probability, and the vertical axis represents the travel cost. This assumption can almost be achieved in public life. Hence, its evaluation effect is more consistent with reality than the other three improved models, and the accounting results are more accurate [16].

The essence of general ecotourism and eco-birdwatching tourism is the consumption of ecological products, such as a good ecological environment and abundant bird resources, by tourists. Ecological products are a kind of public good whose value cannot be determined by market transaction prices. The travel cost method and its derivative models are widely used in evaluating recreation sites and recreation resources in academic circles because of their classical nature when measuring the value of public goods and resources [17]. There are two main methods. One is to combine it with the contingent valuation method (CVM) [18], such as in Song Qing (2018) [19], which combines TCM with CVM and the direct income method to evaluate the recreation value of urban parks in Shanghai, in which TCM reflects the characteristics of tourists and calculates the indirect value of recreation value, while CVM and the direct income method are combined to calculate the immediate recreation value. Clara et al. (2018) [20] estimated the values assigned by recreational visitors to the coastal lagoon at the Ria de Aveiro, Portugal, using both the TCM and the CVM. Yang Shuhao et al. (2019) [21] used TCIA and CVM to evaluate the recreation value of Guanmenshan Forest Park in the context of rural revitalization, and Liang Ping et al. (2016) [22] applied ZTCM and CVM to evaluate the recreation value of Qinghai Lake Scenic Area. The other approach is to use the travel cost method and its derivative models for research. Wang Lifang et al. (2015) evaluated the recreation value of Lishan Forest Park in Shaanxi Province based on TCM [23]. Mayer and Woltering (2018) estimated the recreational ecosystem services of 15 German national parks using zonal travel cost models [24]. Zhao Jianbo et al. (2017) evaluated the recreation value of the main tourist spots in Lhasa City with the regional travel cost method and calculated that its recreation value was 87,567 billion Yuan [25]. Sinclair et al. (2020) used the crowdsourced travel cost method (CTCM) to obtain the welfare estimates of German national parks [26]. Deng Maotao et al. (2020) improved the public cost-sharing model of multi-destination travel and applied it to the recreation value assessment of Ruoergai County [27]; Cai Yinying and Zhang Anlu (2008) applied ZTCM and ITCM to assess the recreation value of farmland landscape [28]; Li Na and Pan Wen (2010) applied TCIA to determine the recreation value of Shennongjia Nature Reserve [29]; Mou Xuejie et al. (2019) evaluated the leisure tourism value of seven A-level natural landscapes in Yanqing District based on TCIA [30]; Zhou Jinying et al. (2021) assessed the ecotourism service value of the “One Village, Ten Thousand Trees” project in Kecheng District of Quzhou City based on TCIA [31]. The research content is mainly divided as follows. First, the study area is compared and analyzed in time and space. Xiao Jingyi et al. (2020) selected Huzhu Beishan Natural Park in Qinghai Province as the study area, evaluated its recreation value, compared it in time and space, and found its recreation value had nearly doubled from 2007 to 2018, and the forested district makes the most significant contribution to the tourism resources of the park in 2018 [32]. The second approach is to create a comparative analysis of the study area, such as that of Hao Weigang (2007), which estimated that the recreation value of Wuliangsuhai National Nature Reserve in 2005 was 26.0471 million Yuan, which was evaluated by group TCIA [33]. Wang Yong et al. (2020) used the improved travel cost-sharing model to calculate the recreation value of Wenchuan County and figured that the recreation value of the study area was 8.325 billion Yuan, and the per capita recreation value was 1387 Yuan, of which the travel cost was 6.669 billion Yuan [34].

In the previous literature on the evaluation of recreation sites and recreation resources by the travel cost method, most were based on their own research focus and data characteristics, or selected and innovated using derivatives of the travel cost method, or uses the combination of the travel cost method and other methods such contingent valuation. However, there are few studies on calculating and comparing the recreation value of special ecotourism forms and traditional ecotourism forms. At the same time, compared with other forms of ecotourism, birdwatching tourism is more unbalanced and restricted by regional endowments, and its establishment is highly dependent on the richness of species and quantity of bird resources. Therefore, birdwatching tourism research focuses more on its regional nature to provide guidance for developing the local bird-viewing tourism industry according to local conditions.

Based on this, this paper applies travel cost interval analysis (TCIA) to calculate the recreation value of eco-birdwatching tourism in Mingxi County and compares it with the recreation value of the local general eco-birdwatching industry to explore whether the development of the eco-birdwatching industry will achieve higher ecosystem service value from an empirical point of view.

## 3. Materials and Methods

### 3.1. Data and Materials

#### 3.1.1. Study Area

**Figure 1:**
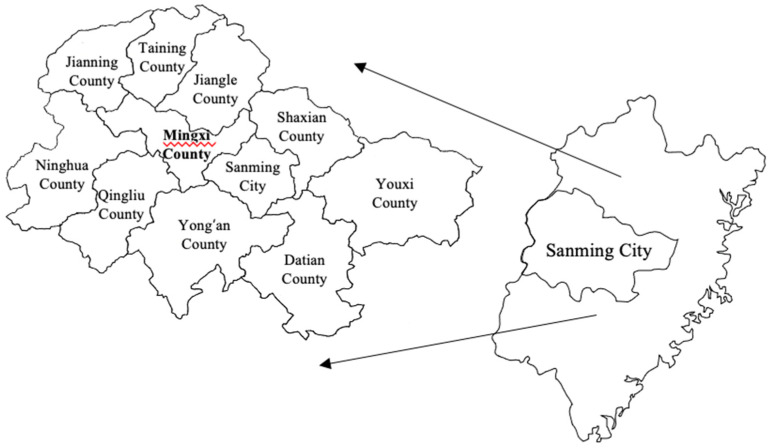
Study area.

We selected China’s Mingxi County, one of three birdwatching resorts and one of the areas with the highest abundance of bird resources in China. In terms of geography, Mingxi County is in the northwest of Fujian Province, China, and the central part of Sanming City, with a total area of 1730 square kilometers and 82% forest coverage. It is located on the international migratory route from East Asia to Australia and is the only way for migratory birds to cross Fujian and Jiangxi provinces. It is also an important resting place for migratory birds and a breeding place for summer migratory birds in eastern China (Figure 1).

Based on effective ecological bird biodiversity protection, the habitat is in good condition. In terms of biodiversity, there are more than 100 species and millions of migratory birds, such as swans, Mandarin ducks, cormorants, and snipes, passing through Mingxi every year. 316 species of wild birds have been found in this region; there are many endangered and rare birds, including four species of “panda in birds” *Tragopan caboti*, *Syrmaticus ellioti*, *Ciconia boyciana,* and *Mergus squamatus*, which are protected by the state at the first level, and 34 species of *Aix galericulata*, *Lophura nycthemera,* and *Lophura nycthemera* protected by the state at the second level. In addition to these nationally protected birds, it also has *Rostratula benghalensis*, *Eurystomus orientalis*, *Melanochlora sultanea*, *Merops viridis*, *Merops viridis*, *Aethopyga latouchii,* and other bird species with great ornamental and protection value as local star bird species [35]. At present, eco-birdwatching tourism in this area has entered the initial stage of development.

**Figure 2:**
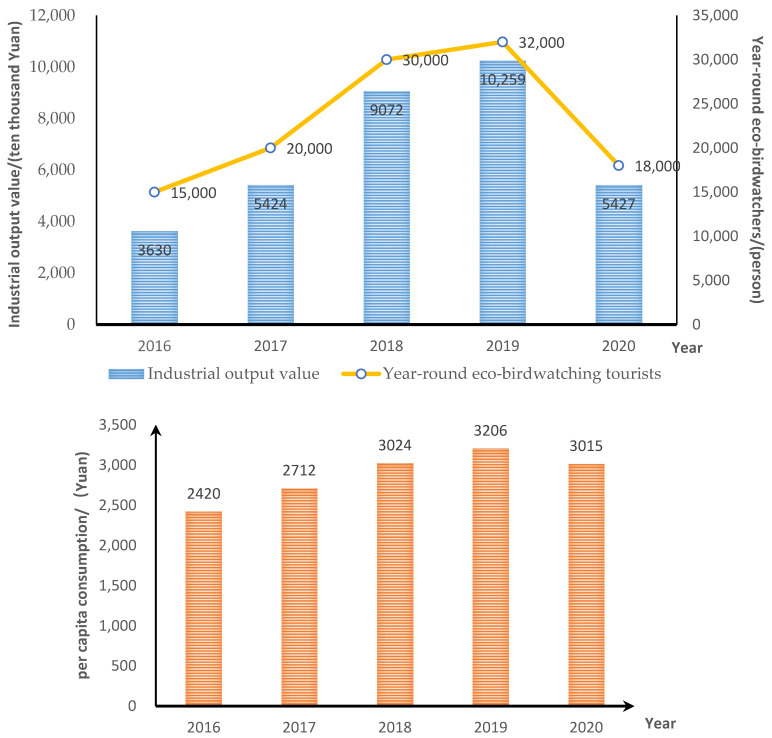
Numbers and output value for eco-birdwatching tourism in Mingxi County.

Through vigorous development of the eco-birdwatching industry, it has successfully launched its popularity with birders and gained visibility, attracting many birdwatching tourists, thus leading to the development of local tourism, tourism services, and other related industries (such as printing and advertising). The trend of the output value of the ecological bird watching industry is generally in line with the trend in annual tourists for eco-birdwatching, while the trend of per capita consumption is also increasing year by year, but the growth trend is slow. Since the eco-birdwatching industry sprouted in 2016, the annual number of eco-birdwatchers has grown every year. However, the sudden outbreak of COVID-19 at the start of 2020 has caused the local birdwatching tourism industry to suffer heavy losses, and construction projects have stagnated for a time [36], with the eco-birdwatchers’ number dropping from 32,000 in 2019 to 18,000 in 2020. However, by the early spring of 2021, with the gradual stabilization of the domestic COVID-19 epidemic and the gradual liberalization of travel from other municipal and provincial areas, even nationwide, the local ecological bird-watching industry has gradually recovered in 2021 (Figure 2).

There are many highly similar birdwatching attractions in China, so selecting such a typical birdwatching resort situated along migratory routes can reflect the differences in value between eco-birdwatching tourism and general ecotourism. Therefore, our study is highly replicable.

There are many birdwatching destinations globally, and Mingxi County is just one of them, but is famous in China. Each birdwatching resort attracts different bird-watching tourists with varying characteristics because of its different star bird species. Therefore, the comparative study of ecological birdwatching and general ecotourism can be expanded in the future to study the universality of this law.

For locals, the protection of wild animals means that they may have lost the opportunity to develop the local economy through industrialization at the same time, so the development of ecotourism, especially eco-birdwatching tourism, based on good local habitat conditions, is a way to compensate the local economy, but also a way to transform the value of ecosystem services. However, the development of eco-tourism needs to be adapted to local conditions and diversity characteristics, so the future should employ a more targeted research approach.

#### 3.1.2. Data Collection

Survey data were collected from Mingxi County, China in March–April 2021. To ensure that the questionnaire questions were set up more rationally, the samples showed more coverage, and the answers were more realistic, our survey team consulted a large amount of secondary information, including the Internet and regional statistical yearbooks, especially the distribution and overall planning of regional birdwatching resorts, and conducted many discussions with the staff of wildlife protection stations and senior birdwatchers to improve our pre-research questionnaire and research plan. Six graduate students conducted a preliminary survey in October–November 2020, and the feedback was used to understand whether the intervals and choices in the design of our questionnaires were reasonable and consistent with reality. The eco-birdwatchers who engaged in birdwatching activities in birdwatching spots and general ecotourists in scenic ecotourism spots here were selected as the survey objects. The content of our questionnaire includes the interviewee’s characteristics, tourism purpose, and travel costs for ecotourism.

The survey objectives and questions were explained to the participants to minimize potential miscommunication when administering the questionnaire. Once the participants had finished the questionnaire, they received thank-you gifts, such as bottles of water. More than 95% of tourists were able to complete the survey with the help of investigators. After the preliminary survey, we synthesized all the opinions and suggestions from experts and objectives and determined the final questionnaire.

During the survey period, the epidemic still affected the situation, and there were certain restrictions on ecotourists, especially professional birdwatchers. Therefore, our research team adjusted and optimized the survey plan based on the preliminary results and current conditions, using a combination of paper questionnaires distributed on the spot and electronic questionnaires distributed online to birdwatchers and members of birdwatching associations to launch the main survey in spring 2021. The paper version of the questionnaire was divided into two parts: the questionnaire for general ecotourists and that made especially for eco-birdwatchers. The electronic version of the questionnaire was for professional birdwatchers who have recently conducted eco-birdwatching in Mingxi County. Our data collection team comprised six graduate students with rich experience in social surveys. Like the participants who engaged in the paper version, those interviewees who completed the online questionnaire received digital currency worth 2 yuan as a thank-you gift.

After collecting the electronic questionnaire data, we checked the questionnaires’ quality individually by computer, and the invalid questionnaires due to incomplete filling in and careless answering attitude were eliminated. Finally, we collected 328 valid questionnaires in total, including 226 paper questionnaires and 102 electronic questionnaires, involving 212 ordinary tourists’ questionnaires and 116 ecological birdwatchers’ questionnaires (Table 1).

### 3.2. Model Construction of TCIA

TCIA is calculated according to each tourist’s actual travel cost and characteristics. All travel costs are calculated through offline and online questionnaires. Travel costs (TC) include explicit expenses and implicit expenses. Explicit expenses refer to the total expenses spent by tourists on travel, including transportation, accommodation, catering, entrance fees, guide and birdwatching seats, souvenirs, and agricultural products. Implicit expenses include time opportunity costs, which are included via the calculation of one third of the wage rate per unit time.

#### 3.2.1. Travel Cost

The travel cost of the eco-birdwatcher includes the individual transportation cost TC_transport_, the personal accommodation cost TC_accommodation_, the total cost for individual food and beverages TC_food_, the individual ticket cost TC_ticket_, the eco-birdwatching service cost TC_visiting_, including the guide fees and the seat fees, the cost of purchasing agricultural products and souvenirs TC_shopping_ and the opportunity cost TC_time_. TC is the total travel cost. Therefore, for the eco-birdwatchers’ group, the travel cost formula is as follows:

TC = TC_transport_ + TC_accommodation_ + TC_food_ + TC_ticket_ + TC_visiting_ + TC_shopping_ + TC_time_, (1)

Compared with the travel cost of ecological birdwatchers (Formula (1)), the total travel cost of ordinary tourists (Formula (2)) does not include TC visiting, which is unique to eco-birdwatching tourism. For the general ecotourists’ group, the travel cost formula is as follows:

TC = TC_transport_ + TC_accommodation_ + TC_food_ + TC_ticket_ + TC_shopping_ + TC_time_, (2)

The opportunity cost is calculated according to one third of the wage rate, and the monthly disposable income Income_monthly_, monthly salary days Days_paid-monthly_, average weekly working hours Working-hours_weekly_, visiting hours Hours_visiting_ and total round-trip traffic hours Hours_transport,_ required for the calculation of the wage rate, are all included in the questionnaire to collect the corresponding data.

Unit time cost = 1/3 × Income_per-hour_, (3)

Income_per-hour_ = Income_monthly_/Days_paid monthly_ × (Working-hours_weekly_/6), (4)

The number of paid days per month is calculated according to the number of statutory holidays stipulated by the Ministry of Labor and Social Security of China as follows:

Days_paid monthly_ = (365 − 104) days/12 months = 21.75 days, (5)

TC_time_ = Unit time cost × (Hours_visiting_ + Hours_transport_), (6)

#### 3.2.2. TCIA Model

Based on the field survey data of TCIA, the travel costs of the two categories of tourists are divided into different cost ranges to ensure that the travel costs of tourists in each group are similar. Assuming that the total sample sizes of eco-birdwatchers is N_a_, the total travel cost of each visitor is obtained through questionnaire survey, which is divided into different intervals according to the travel expenses: $[C_{a_{0}},C_{a_{1}}]$,$[C_{a_{1}},C_{a_{2}}]$,$[C_{a_{2}},C_{a_{3}}]$,……, $[C_{a_{i}},C_{a_{i+1}}]$ ,……$[C_{a_{n-1}},C_{a_{n}}]$, [$C_{a_{n-1}}$,$C_{a_{n}}$],$[C_{a_{n}},+\infty )$, a total of (n + 1) sets. The number of tourists in each set is $N_{a_{0}}$, $N_{a_{1}}$ , $N_{a_{2}}$ , ……, $N_{a_{i}}$ , ……, $N_{a_{n}}$ . The following assumption is that each eco-birder in the *i* set is willing to take an eco-birding tour when the travel cost is $C_{a_{i}}$, and is also willing to take a tour at a cost less than $C_{a_{i}}$. So, when the travel cost is $C_{a_{i}}$, the travel demand of the sample eco-birdwatchers $M_{a_{i}}$ can be expressed as:

(7) $$M_{a_{i}}=\sum_{i}^{n}N_{a_{j}}\left(i\leq j\leq n\right),$$

(8) $$P_{a_{i}}=\frac{M_{a_{i}}}{N_{a}}\left(0\leq i\leq n\right),$$

$P_{a_{i}}$ is the proportion of N_a_ eco-birdwatchers who are willing to take eco-birdwatching tours at the price of $C_{a_{i}}$. Suppose N_a_ birdwatchers have the same demand for birdwatching tourism. In that case, it can be considered that the probability of each ecological birdwatcher conducting birdwatching tourism at the price of $C_{a_{i}}$ is $P_{a_{i}}$, that is:

(9) $$P_{a_{i}}=Q_{a_{i}},$$

In this formula, $Q_{a_{i}}$ is the birdwatching tourism willingness demand of each eco-birdwatcher at a price of $C_{a_{i}}$. According to the previous literature [37,38], linear and logarithmic regression fitting methods are selected respectively, i.e., the ecotourism willingness demand curve of a single ecotourist, and the function with higher R^2^ is taken as the ecotourism demand curve. So the birdwatching tourism willingness demand curve of a single eco-birdwatcher can be obtained by regression fitting on $C_{a_{i}}$ with $Q_{a_{i}}$, which is defined as the eco-birdwatching tourism forest-visiting rate curve. Assuming that the total sample number of general ecotourists is N_b_, in the same way the tourism willingness demand curve of a single general ecotourist is obtained by regression fitting with the data points of $C_{b_{i}}$ and $Q_{b_{i}}$, which is defined as the forest-visiting rate curve of general ecotourism.

#### 3.2.3. Consumer Surplus

The consumer surplus calculation formula of TCIA is as follows:

(10) $${\mathrm{CS}}_{i}=\int_{C_{i}}^{\infty }Q\left(C\right)\mathrm{dc},$$

where CS_i_ represents the consumer surplus of each tourist in the cost interval *i*. C_i_ represents the lower limit of the travel cost in the cost interval *i*. Q(C) represents the travel intention demand curve for a single tourist. The total consumer surplus of N sample tourists is:

(11) $$\mathrm{SCS}=\sum_{i=0}^{n}N_{i}{*\mathrm{CS}}_{i},$$

The total travel costs of the sample visitors were:

(12) $$\mathrm{STC}=\sum {\mathrm{TC}}_{i},$$

The total recreation value (RV) of ecotourism in Mingxi County is expressed as follows:

(13) $$\mathrm{RV}={\mathrm{RV}}_{a}+{\mathrm{RV}}_{b},$$

(14) $${\mathrm{RV}}_{a}=\left({\mathrm{SCS}}_{a}+{\mathrm{STC}}_{a}\right)/N_{a}\times {\mathrm{TN}}_{a},$$

(15) $${\mathrm{RV}}_{b}=\left({\mathrm{SCS}}_{b}+{\mathrm{STC}}_{b}\right)/N_{b}\times {\mathrm{TN}}_{b},$$

where RV_a_ represents the recreation value of the eco-birdwatching industry in Mingxi County. RV_b_ represents the recreation value of the general ecotourism industry in Mingxi County. SCS_a_ represents the total consumer surplus of the eco-birdwatching sample. SCS_b_ is the total consumer surplus of the general ecotourist sample. STC_a_ represents the total travel cost of the eco-birdwatcher sample, and STC_b_ represents the total travel cost of the general tourist sample. N_a_ represents the total number of eco-birdwatcher samples; N_a_ = 116. N_b_ represents the number of general tourist samples; N_b_ = 212. TN_a_ represents the annual flow of eco-birdwatchers here, and TN_b_ represents the annual flow of general ecotourists in this region.

## 4. Results and Discussion

### 4.1. Descriptive Statistical Analysis of Tourists Demographics

This study performed descriptive statistical analysis on tourists’ social, economic, and personal characteristics data. The results of statistical analysis among eco-birdwatchers and general ecotourists are shown below (Table 2).

According to the survey results, the proportion of male and female respondents in general ecotourists is almost equal (54.72 percent for men and 45.28 percent for women), while the sex ratio is very different with professional birdwatchers. Most eco-birdwatchers are men, which may be related to men preferring to travel in the mountains, by water and close to nature more than women. People between the ages of 50 and 59 are the indispensable main force in both eco-birdwatching and general ecotourism activities because they have gone through the struggle of their younger working period and are now at the stage of waiting for retirement. Their working time is significantly reduced compared to young people, and their children are usually adults who are independent already. In eco-birders, the proportion of people aged 50 to 59 is significantly higher than that of other age groups. Eco-birdwatching is still an “older person’s activity” in the general stereotype, while the older population is generally more likely than the youngers to watch birds, so they are the mainstream of the birdwatching population. At the same time, people under 20 years of age and over 60 years of age may be affected by their physical condition, education level, and cognitive situation. The low level of participation in the questionnaire filling process may also affect the number and distribution of these age groups to some extent.

The educational level of eco-birdwatchers is generally higher than that of the general ecotourists. The number of eco-birdwatchers with primary school education and below, junior high school education and high school education level is significantly lower than that of ordinary ecotourists, while in the number of people with specialized and higher education, eco-birdwatchers (72.41%) are represented significantly more than the general tourists (65.57%). This may be because eco-birdwatching tourism has a higher threshold than general ecotourism, which requires birdwatchers to learn about bird habits, activity patterns, appearance, sound, and other relevant knowledge and birdwatching skills, so there are higher requirements for individuals in terms of cognitive level, learning ability, economic level, etc. At the same time, the more educated population is more likely to be exposed to birdwatching knowledge because of their learning ability, higher cognitive level, and exposure to bird-watching knowledge. So their probability of engaging in bird-watching activities is higher than that of less-educated people. This reflects the generally higher education level of eco-birdwatchers than general ecotourists.

In our study, the average monthly disposable income of individuals used to measure the actual income level of respondents, including personal wage income, stock funds, insurance, and other financial, housing and other property income, is the total disposable income of individuals after tax. This is not only a reflection of personal ability to make money, but also a reflection of personal living standards. The monthly disposable income of eco-birdwatchers is more than 4000 to 6000 yuan, and the proportion in the income segment above 6000 yuan is significantly higher. While most general ecotourists are in the 2000-to-4000-yuan income segment, their income distribution is mainly at a lower level from the overall point of view. Eco-birdwatchers who are about to retire or have retired are higher in number, so the proportion working less than 20 h per week (35.34%) is higher than otherwise (25.94%).

### 4.2. Forest-Visiting Rate Curve

#### 4.2.1. Forest-Visiting Rate Curve for Eco-Birdwatching Tourism

Based on the questionnaire data, this paper considers the distribution of sample points and the interval span. It divides the travel expenses of ecological birdwatchers into 34 consumption intervals according to 500 Yuan intervals. The travel demand of a single birdwatcher $Q_{a_{i}}$ and the lower limit of the corresponding division interval $C_{a_{i}}$ are regressively fitted. The linear regression equation is C_a_ = −18526 Q_a_ + 12,964. The determination coefficient R^2^ = 0.6185, and Sig = 0.02. The logarithmic fitting equation is C_a_ = −4380ln (Q_a_)—1640.8, with the determination coefficient R^2^ = 0.9347 and Sig = 0.000. Currently, the Sig value is less than 0.05, indicating significance at the 5% level. The goodness of fit is greater than 0.9 and very close to 1, indicating that the fitting effect of the logarithmic form is better than that of linear regression. Therefore, the logarithmic fitting curve is selected as the eco-birdwatching tourism forest-visiting rate curve, which explains the relationship between the birdwatching tourism demand rate of a single ecological birdwatcher and the travel cost. Therefore, the curve of eco-birdwatching tourism forest-visiting rate in Mingxi County is:

(16) $$C_{a}=-4380*\ln\left(Q_{a}\right)-1640.8,$$

So the total consumer surplus of the sample of ecological birdwatchers in Mingxi County is 200,370.39 Yuan, and the total travel cost of the sample eco-birdwatchers is 484,889.76 Yuan.

#### 4.2.2. Forest-Visiting Rate Curve for General Ecotourism

In the same way, the travel expenses of general ecotourists were divided into 33 consumption intervals according to 100 Yuan intervals. The linear regression equation is C_b_ = −6333.8 Q_b_ + 4148.6. The determination coefficient R^2^ = 0.2594, and Sig = 0.2. The fitting effect is poor. The equation for the logarithmic regression fit curve is C_b_ = −2310ln (Q_b_) − 2590.3, with a coefficient of determination R^2^ = 0.7848 and Sig = 0.01. Currently, the Sig value is less than 0.05, indicating that it is significant at the level of 5%. The goodness of this fit value is greater than 0.7, indicating that the logarithmic fitting effect is better than the linear fitting effect, so the logarithmic fitting curve is selected as the ecotourism willingness demand curve of a single general eco-tourist. That is to say, the curve equation of general ecotourism forest-visiting rate in Mingxi County is as follows:

(17) $$C_{b}=-2310\ln\left(Q_{b}\right)-2590.3,$$

So, the total consumer surplus of the general eco-tourists sample is 124,049.02 Yuan, and the total travel cost of the general eco-tourists sample is 197,553.33 Yuan.

### 4.3. Differences in Forest-Visiting Rate Demand Curves

From a comparison of these two demand curve equations (Formulas (16) and (17)) and their corresponding figures, it can be found that there is a big difference between the forest-visiting rate curves of eco-birdwatchers and general eco-tourists, so the difference is further explained from two perspectives of mathematical derivation and statistical testing.

#### 4.3.1. Mathematical Derivation

The demand curve of the eco-birdwatching tourism forest-visiting rate in Mingxi County (Formula (16)) and the curve of the general ecotourism forest-visiting rate (Formula (17)) are derived respectively, and the following results are obtained:

(18) $$Q_{\mathrm{eco}-\mathrm{birdwatcher}}^{’}\left(C\right)=-\frac{1}{4380}e^{-\frac{1640.8}{4380}-\frac{C_{i}}{4380}},$$

(19) $$Q_{\mathrm{general}\;\mathrm{ecotourist}}^{’}\left(C\right)=-\frac{1}{2310}e^{-\frac{2590.3}{2310}-\frac{C_{i}}{2310}},$$

where C_i_ ∈[0,+∞). When $Q_{\mathrm{eco}-\mathrm{birdwatcher}}^{’}=Q_{\mathrm{general}\;\mathrm{ecotourist}}^{’}$, C_i_ = −522.65. $Q_{\mathrm{eco}-\mathrm{birdwatcher}}^{’}$ with $Q_{\mathrm{general}\;\mathrm{ecotourist}}^{’}$, in C_i_ ∈[0,+∞) is on the monotony of increment. When C_i_ ∈[0,+∞), $Q_{\mathrm{eco}-\mathrm{birdwatcher}}^{’}\left(C\right)\;$> $Q_{\mathrm{general}\;\mathrm{ecotourist}}^{’}\left(C\right)$ always set up. This shows that the marginal cost of a single eco-birdwatcher is higher than that of a general ecotourist, so the development of eco-birdwatching tourism plays a more prominent role in promoting the local economy than general ecotourism.

**Figure 3:**
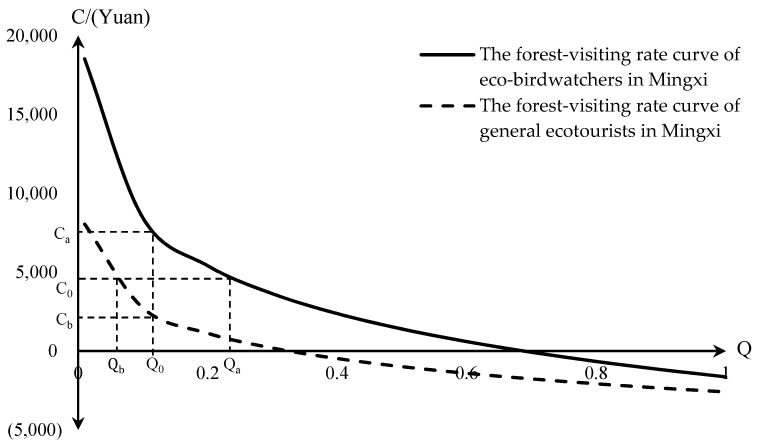
The demand curve of eco–birdwatchers and general ecotourists’ willingness to travel.

Q represents the travel intention demand of individual tourists or the probability of their travel, which can also be regarded as the proportion of tourists to the total number of tourists at the corresponding cost level. It can be seen clearly from Figure 3 that the overall curve of eco-birdwatching forest-visiting rate is above the curve of the general ecotourism forest-visiting rate. When the same price level C_0_ (C_0_ ≥ 0) is taken, Q_a_ − Q_b_ = $e^{-\frac{1640.8+c}{4380}}-e^{-\frac{2590.3+c}{2310}}\;$> 0 is always set up on C_i_ ∈[0,+∞), i.e., Q_a_ > Q_b_ is always true on C_i_ ∈[0,+∞), which indicates that the willingness of eco-birdwatchers to watch birds is stronger than that of general ecotourists at the same cost level. When the same demand level Q_0_ is taken, C_a_ − C_b_ = −2070 * LN (Q) + 949.5 monotonically decreases on Q_i_ ∈[0,1] and is always greater than 0, indicating that the cost of a single eco-birdwatcher is higher than that of a single general ecotourist at the same level of ecotourism willingness demand.

#### 4.3.2. Independent-Samples *t*-Test

To determine whether there is a significant difference in various travel costs between eco-birdwatchers and general ecotourists, the independent-samples *t*-test is used to set the dummy variable according to the interviewees’ identity so that general ecotourists = 0 and eco-birdwatchers = 1. It is assumed that there is no significant difference in various travel costs between the two groups. The test results are shown in Table 3 below.

In the independent-samples *t*-test (Table 3), the significance level of the two groups’ F tests is 0.000, less than 0.05, i.e., the variance is not homogeneous; when the variance is not homogeneous, the significance level of the corresponding *t-test* is 0.000, less than 0.05, i.e., the original hypothesis is rejected, and it is considered that there is a significant difference in travel expenses between general ecotourists and eco-birders. The significant level of the F test of the accommodation fee is 0.308, which is greater than 0.05, and the variance is homogeneous; when the variance is homogeneous, the significant level of the corresponding *t-test* is 0.110, which is greater than 0.05, the original hypothesis is accepted, and there is no difference in accommodation fee between general ecotourists and eco-birders. Similarly, there are significant differences in the cost of catering, tickets, shopping, time cost, and total travel cost between the two.

From the above calculation, we can see the total consumer surplus and total travel expenses of 116 eco-birdwatchers and 212 general ecotourists, and further calculate the per capita consumer surplus, various travel expenses, and total expenses:

(20) $${\mathrm{ATC}}_{a}=\frac{\sum {\mathrm{TC}}_{a}{}_{i}}{N_{a}},$$

(21) $${\mathrm{ATC}}_{b}=\frac{\sum {\mathrm{TC}}_{b}{}_{i}}{N_{b}},$$

where N_a_ represents the total number of samples of eco-birders, i.e., N_a_ = 116. N_b_ represents the total number of samples of general ecotourists, i.e., N_b_ = 212. ${\mathrm{TC}}_{a}{}_{i}$ represents the total travel cost of the sample ecological birdwatcher, while ${\mathrm{TC}}_{b}{}_{i}$ represents the same cost of sample eco-birders. TC, TC_transport_, TC_accommodation_, TC_food_, TC_ticket_, TC_visiting_, TC_shopping_ and TC_time_ are respectively substituted into the total travel cost for calculation. ATC_a_ represents the per capita travel expenses of eco-birders according to the substituted ${\mathrm{TC}}_{a}{}_{i}$. Similarly, ATC_b_ represents the per capita travel expenses of general ecotourists based on the substituted travel expenses. The calculation of consumer surplus per capita is also similar:

(22) $${\mathrm{ACS}}_{a}=\frac{{\mathrm{SCS}}_{a}}{N_{a}},$$

(23) $${\mathrm{ACS}}_{b}=\frac{{\mathrm{SCS}}_{b}}{N_{b}},$$

The calculation results are shown in Table 4 below.

It can be seen from Table 4 that the average total travel cost of general ecotourists is 193.91 Yuan, and the average consumer surplus is 105.94 Yuan, while the average total travel cost of eco-birders is 949.14 Yuan, and the corresponding average consumer surplus is 174.57 Yuan. Moreover, the per capita travel costs of general ecotourists are lower than those of eco-birders. Among them, the higher travel expenses of eco-birders may be related to the longer average distance between their geographical sources and Mingxi County. Overall, there is little difference in per capita accommodation cost, and the statistical test results show no difference between the two because the accommodation conditions here are still limited. Although the standards of homestays and hotels are slightly different, the price difference is not large, and eco-birders (especially senior birders) have very low requirements for accommodation conditions and even bring their own tents and sleeping bags. Similarly, the difference in per capita food and beverage expenses is slight, but the *T*-test results show a significant difference between the two for this item. Although the food and beverage prices here are mainly farm dishes and Hakka dishes, birdwatchers have a wider catering choice, from five-star hotel standard meals to village farmhouse entertainment, because of their high income. While most general ecotourists are local, most of their catering is solved by themselves at home. Eco-birdwatchers spend 178.15 Yuan per person on birdwatching guide fees and seat fees; in addition, their per capita time cost is nearly ten times that of general ecotourists, which is related to the fact that eco-birdwatchers generally have higher wages and longer travel time than general ecotourists.

### 4.4. Results of the Recreation Value of Eco-Birdwatching

According to the above calculation, SCS_a_ = 200,370.39 Yuan, STC_a_ = 484,889.76 Yuan. SCS_b_ = 124,049.02 Yuan, STC_b_ = 197,553.33 Yuan. According to the statistics and estimation of the relevant managers of the Forestry Bureau of Mingxi County, the annual flow of eco-birdwatchers is 32,000, and the annual flow of general ecotourists is 120,000. Substituting them into the above Formulas (13)–(15), we can get RV_a_ = 189,037,282.76 Yuan, RV_b_ = 182,039,066.04 Yuan. The annual social, economic, and ecological benefits realized per capita by general ecotourism and eco-birdwatching are calculated in Table 5 below.

Ecotourism is essentially a process of consuming the forest’s ecological products in the market, and the rich bird resources in Mingxi County are essentially a special ecological product, while the eco-birdwatchers are its consumers. We divided the ecotourism industry in Mingxi County into two parts: the eco-birdwatching industry and the general ecotourism industry, so its ecological benefits can be indirectly calculated by the ecological benefits of these two parts. The ecological benefits are transformed into social benefits and economic benefits in the process of ecotourism. Among them, the social benefit is represented by consumer surplus, which is the travel cost those travelers are willing and able to pay, but the actual payment is less than this part; that is, the benefit obtained by birdwatchers without actual payment is essentially a kind of social welfare. The greater the consumer surplus, the greater the social benefit it achieves. The economic benefit is essentially a kind of manager’s income, which is formed by multiplying the unit price of all goods or services consumed by travelers by the quantity. For example, the income is obtained by providing travelers with transportation, catering, accommodation, birdwatching, garden, and peripheral entertainment services. In the TCIA method, the sum of travelers’ travel expenses is the value of this part of the economic benefit.

Therefore, Mingxi County has achieved a high ecosystem service value realization and ecological product value transformation through its ecotourism industry. The per capita recreation value of eco-birdwatching is 3.9 times that of general eco-tourism, its per capita social benefit is three times that of general ecotourism, and its per capita economic benefit is 4.5 times that of general ecotourism. The ecological bird-watching industry has a higher marginal value than general eco-tourism. It has higher social, economic, and ecological benefits, bringing a higher level of development for the local tourism industry.

From the above calculation results, the total consumer surplus of 116 interviewed eco-birders is higher than that of the 212 interviewed general ecotourists, and the entire travel cost of the sample is also higher than that of the interviewed general tourists. Although the number of general ecotourists is about four times that of eco-birders at the current scale, the annual ecological benefits realized by general ecotourism are still slightly lower than those realized by eco-birdwatching tourism. This shows that compared with traditional ecotourism, the development of eco-birdwatching has higher marginal economic benefits, social benefits, and ecological benefits, and because birdwatching loyalty will increase its tourism’ stickiness, the revisit rate and tourism frequency are higher, so it is a sustainable tourism form in the long run. From the perspective of economic promotion and social benefits, this also shows that the Sanming Municipal Government has correctly positioned the birdwatching industry as the development strategy for Mingxi County.

Only when there is a suitable habitat for birds to survive can there be birds with high abundance, and only when there is a high abundance of birds can there be birdwatching behavior in Mingxi, so the consumer surplus (social benefit) of each eco-birdwatcher and the economic benefit based on his actual payment can be formed, and “ecology is economy” be realized by eco-birdwatching as a means of ecological value transformation. The calculated RV is the conversion value of ecological products produced by ecotourism. The social and economic benefits of the eco-birdwatching industry can be achieved by promoting birdwatching in Mingxi County and the arrival of eco-birdwatchers. It is precisely because of the good ecological environment that the complete ecological service function can be brought into play. On this basis, the development of the eco-birdwatching industry is essentially “turning green mountains into golden mountains and silver mountains”, which also gives local ecological benefits a possibility of realization.

Ecotourism is essentially the process of tourists’ consumption of ecological products, which can realize the value conversion of local ecosystem recreation value through eco-birdwatching and general ecotourism, and this consumption process affects the realization of recreation value. This study shows that eco-birdwatching is the most powerful way to realize the value transformation of local ecological value in a birdwatching resort, which has the advantages of sustainability, high marginal value, and a stable audience compared with the traditional ecotourism industry. Therefore, in the future, similar birdwatching resorts should firmly develop their eco-birdwatching industry to stimulate the county’s economy and promote the upgrading of other related industries, to truly achieve poverty alleviation and rural revitalization.

How to transform ecological advantages into industrial advantages is an urgent issue for each specific regional unit to plan and implement under this background. How to realize the coordination of protection and development, and how to consider both ecological benefits and social and economic benefits, are essential propositions that need to be discussed urgently in every ecotourism development area, and the actual realization of “ecological industrialization, industrial ecology” is the key to solving this contradiction. For a birdwatching resort, how to better industrialize birdwatching and promote the development of surrounding industries and county economy by birdwatching, on the premise of protecting ecology and ensuring that birds are minimally disturbed by human beings in their natural state, are urgent topics to be discussed.

## 5. Conclusions

In this study, travel cost interval analysis was used to evaluate the recreation value of eco-birdwatching and the general ecotourism industry. In designing the questionnaire and the data collection, we identified each interval of selection based on the results’ distribution of a preliminary survey in October–November 2020 and finally including personal characteristics, tourism purpose, and travel costs to compare the differences between eco-birdwatching and general ecotourism in terms of each expense and its probability factors in Mingxi County. We tried to analyze these differences from an economic point of view by fitting forest-visiting demand curve and necessary mathematical deduction, as well as independent-samples *T*-test, to check which cost will lead to differences. Additionally, descriptive analysis of ecotourists from social, economic, and personal standpoints was also complemented by explanation of differences.

First, the recreation value of the ecological bird-watching industry is about 189 million yuan. In comparison, that of general ecotourism is about 182 million yuan with more than three times the number of birders. The recreation value of eco-birdwatchers per capita is 5688.72 yuan. The per capita recreation value of eco-birdwatching is 3.9 times that of general eco-tourism, its per capita social benefit is three times that of general ecotourism, and its per capita economic benefit is 4.5 times that of general ecotourism. For areas focused on biodiversity conservation and owning rich bird species, developing ecotourism featuring ecological birdwatching is a more effective way to enhance the local economy and transfer natural capital and ecosystem services into human well-being.

Second, compared with general ecotourists, the per capita travel costs of eco-birdwatchers are higher, and there were significant statistical differences in the expenses of catering, tickets, shopping, opportunity costs, and total travel expenses between these two groups. Birdwatching destinations such as Mingxi county should focus on how to increase birdwatching satisfaction and tourist‘ stickiness via hardware facilities and ancillary services to promote stay-days and willingness to revisit.

Third, in comparison to general ecotourists, the marginal cost of an individual eco-birdwatcher is higher, and the travel intention of an eco-birdwatcher is more robust at the same cost level. The cost of a single eco-birdwatcher is higher at the same travel intention demand level.

Fourth, eco-birdwatchers were mainly middle-aged and elderly men, among which retired people accounted for a large proportion. Their income level was higher, and average weekly working hours were less.

In short, ecological bird-watching tourism has higher recreation value than general tourism. Therefore, these migratory areas can focus on developing eco-birdwatching tourism instead of general ecotourism, not only to avoid the negative impact of large-scale tourism but also to achieve higher ecological value transformation.

## Figures and Tables

**Table 1:** Data collection.

Questionnaire Type	Version	Spot	Number
general ecotourists	total		212
	paper version	Tsui Zhuyang Crater Geological Park	31
		Drip Rock Red Scenic Area (Yuxu Hole)	78
		Ziyun Village	81
		Danshang Village	10
		Xiabian Village	4
		Xiaojiashan Village	8
eco-birdwatchers	total		116
	paper version	Ziyun Village	11
		Xiaojiashan Village	3
	electronic version		102
total			328

**Table 2:** Sample structure distribution.

Basic Features	Classification	Eco-Birdwatchers (Person)	General Ecotourists (Person)
Sex	Male	90	116
	Female	26	96
Age (years)	≤19	0	5
	20–29	3	42
	30–39	14	71
	40–49	21	38
	50–59	54	45
	≥60	24	11
Educational Level	Primary school and below	0	3
	Junior middle school	4	34
	High school (vocational)	8	36
	Specialty	37	54
	Undergraduate	36	75
	Master	9	8
	Doctor and above	2	2
Current personal monthly income (Yuan)	≤2000	6	25
	2001–4000	19	78
	4001–6000	34	60
	6001–8000	15	27
	8001–10,000	16	6
	10,001–20,000	18	7
	20,001–50,000	6	4
	≥50,000	2	5
Average weekly working hours (h)	0	36	14
	0–20	5	41
	20–40	42	75
	40–60	28	70
	≥60	5	12

**Table 3:** Independent-samples *t*-test of various travel costs for the two groups.

Travel Cost Category	Levene’s Test for Equality of Variance		*t*-Test for Equality of Means	
	F	Sig.	t	Sig.(2-tailed)
TC_transport_	47.450	0.000 ^***^	−5.520	0.000 ^***^
TC_accommodation_	1.043	0.308	−1.602	0.110
TC_food_	2.026	0.156	−4.315	0.000 ^***^
TC_ticket_	128.784	0.000 ^***^	−4.653	0.000 ^***^
TC_shopping_	9.606	0.002 ^***^	−2.457	0.015 ^***^
TC_time_	10.197	0.002 ^***^	−1.881	0.062 ^**^
TC	14.064	0.000 ^***^	−2.938	0.004 ^***^

^***^ indicates that it is significant at a confidence level of 95%; ^**^ indicates that it is significant at a confidence level of 90%.

**Table 4:** Consumer surplus and travel cost per capita for general ecotourists and eco-birdwatchers.

Type	Eco-Birdwatcher	General Ecotourist
ATC_transport_	¥949.14	¥193.91
ATC_accommodation_	¥174.57	¥105.94
ATC_food_	¥274.57	¥153.64
ATC_ticket_	¥42.59	¥2.41
ATC_visiting_	¥178.15	--
ATC_shopping_	¥143.12	¥54.58
ATC_time_	¥4180.08	¥421.38
ATC	¥949.14	¥193.91
ACS	¥174.57	¥105.94

**Table 5:** Annual social, economic, and ecological benefits achieved per capita by ecotourism.

Type	Social Benefits	Economic Benefits	Ecological Benefits
Eco-birdwatching tourism	¥1727.33	¥4180.08	¥5907.42
General ecotourism	¥585.14	¥931.86	¥1516.99

## Data Availability

The data presented in the study are available upon request from the corresponding author. The data are not publicly available because of privacy concerns.
